# Local compartment changes and regulatory landscape alterations in histone H1-depleted cells

**DOI:** 10.1186/s13059-015-0857-0

**Published:** 2015-12-23

**Authors:** Geert Geeven, Yun Zhu, Byung Ju Kim, Boris A. Bartholdy, Seung-Min Yang, Todd S. Macfarlan, Wesley D. Gifford, Samuel L. Pfaff, Marjon J. A. M. Verstegen, Hugo Pinto, Marit W. Vermunt, Menno P. Creyghton, Patrick J. Wijchers, John A. Stamatoyannopoulos, Arthur I. Skoultchi, Wouter de Laat

**Affiliations:** Hubrecht Institute-KNAW & University Medical Center Utrecht, Uppsalalaan 8, 3584 CT Utrecht, The Netherlands; Department of Cell Biology, Albert Einstein College of Medicine, 1300 Morris Park Avenue, Bronx, NY 10461 USA; Program in Genomics of Differentiation, Eunice Kennedy Shriver National Institute of Child Health and Human Development, Bethesda, MD 20892 USA; Gene Expression Laboratory and the Howard Hughes Medical Institute, The Salk Institute for Biological Studies, 10010 North Torrey Pines, La Jolla, CA 92037 USA; Department of Genome Sciences, University of Washington, Seattle, WA 98195 USA; Department of Medicine, Division of Oncology, University of Washington, Seattle, WA 98195 USA

**Keywords:** Histone H1, Chromatin, Chromosome conformation, Hi-C, Epigenomics

## Abstract

**Background:**

Linker histone H1 is a core chromatin component that binds to nucleosome core particles and the linker DNA between nucleosomes. It has been implicated in chromatin compaction and gene regulation and is anticipated to play a role in higher-order genome structure. Here we have used a combination of genome-wide approaches including DNA methylation, histone modification and DNase I hypersensitivity profiling as well as Hi-C to investigate the impact of reduced cellular levels of histone H1 in embryonic stem cells on chromatin folding and function.

**Results:**

We find that depletion of histone H1 changes the epigenetic signature of thousands of potential regulatory sites across the genome. Many of them show cooperative loss or gain of multiple chromatin marks. Epigenetic alterations cluster to gene-dense topologically associating domains (TADs) that already showed a high density of corresponding chromatin features. Genome organization at the three-dimensional level is largely intact, but we find changes in the structural segmentation of chromosomes specifically for the epigenetically most modified TADs.

**Conclusions:**

Our data show that cells require normal histone H1 levels to expose their proper regulatory landscape. Reducing the levels of histone H1 results in massive epigenetic changes and altered topological organization particularly at the most active chromosomal domains. Changes in TAD configuration coincide with epigenetic landscape changes but not with transcriptional output changes, supporting the emerging concept that transcriptional control and nuclear positioning of TADs are not causally related but independently controlled by the locally associated *trans*-acting factors.

**Electronic supplementary material:**

The online version of this article (doi:10.1186/s13059-015-0857-0) contains supplementary material, which is available to authorized users.

## Background

DNA in the eukaryotic nucleus is packaged into arrays of nucleosome core particles that are the basic unit of chromatin [1, 2]. Each nucleosome consists of an octamer of four core histones (H2A, H2B, H3 and H4) around which about 145 bp of DNA is wrapped. Chromatin also contains a fifth histone, the linker histone, usually referred to as H1. H1 binds to nucleosome core particles near the DNA entry/exit position and to the linker DNA between core particles, stabilizing the association of core particles and DNA and facilitating folding of oligonucleosome arrays into compact structures. Mice and humans express 11 H1 subtypes, including H1a to H1e found at varying levels in most cell types, a replacement subtype (H1(0)) generally associated with terminal differentiation and quiescent states, four germ cell-specific H1s (H1t, H1T2, H1LS1, and H1oo) and a less well-studied subtype (H1x) [3–5]. In addition to their different developmental expression patterns and abundance, the amino acid sequences of these H1 subtypes differ significantly. Despite these differences, we [6–8] and others [9–11] found that elimination of any one of several H1 subtypes, and even some pairs of subtypes, does not noticeably affect mouse development. The absence of phenotypes in these mice appears to be due to up-regulation of the remaining subtypes, resulting in maintenance of a normal H1 to nucleosome core particle stoichiometry. However, elimination of three H1 subtypes (H1c, H1d, and H1e) together led to a 50 % reduction in the ratio of H1 to core particles and embryonic lethality. Embryonic stem (ES) cells derived from H1c, H1d, H1e null embryos are viable and also exhibit a 50 % reduction in H1:core histone stoichiometry. They show a decrease in the average spacing between nucleosome core particles of about 15 bp, from ~189 bp in normal ES cells to ~174 bp in the triple H1 knock-out (TKO) ES cells [12]. These TKO ES cells also showed decreased local chromatin compaction and selective changes in gene expression. Importantly, up-regulation of certain imprinted and X chromosome-linked genes were prominent and found to be due to H1-dependent alterations in DNA methylation and H3 histone methylation at the regulatory regions of the affected gene [12–14]. Reduced H1 levels were also found to enable CTCF to bind to normally occluded DNA sequences at some imprinted gene loci [13]. CTCF is a central factor in setting up local chromatin loops and defining structural domains across mammalian chromosomes [15, 16]. Collectively, this suggests that histone H1 may also have an important function in shaping higher order genome structures in vivo, either directly through its capacity to compact DNA or indirectly by controlling the DNA accessibility of chromatin architectural proteins.

To investigate the role of H1 in genome-wide, higher order chromatin structure, we studied the regulatory landscape and overall genome conformation in the H1-depleted TKO ES cells [12]. ES cells show several unique features of nuclear organization compared with somatic cells. For example, ES cells display hypermobility of chromatin proteins, including the core histones and histone H1, indicative of their loosened binding to DNA [17]. Restricting the dynamic state of these core chromatin components compromises the differentiation capacity of ES cells, suggesting that this feature is essential for ES cell identity [17, 18]. ES cells also have an unusually low H1 to nucleosome core stoichiometry: whereas this ratio is typically 0.75 or more in differentiated cells, in wild-type ES cells it is only about 0.5 [19]. In the H1 TKO ES cells, this ratio is further reduced to one histone H1 molecule per four nucleosomes [20]. ES cells also display a distinctively disorganized three-dimensional (3D) genome with particularly inactive chromosomal regions that fail to cluster as efficiently as seen in somatic cells [21]. Here we applied Hi-C [22] and other genome-wide approaches for mapping epigenetic features to compare wild-type and H1-depleted ES cells to better understand how the H1 linker histone impacts the regulatory and 3D landscape of the genome and its consequences on transcription.

## Results and discussion

### Clustered DNA methylation changes in histone H1-depleted ES cells

**Fig. 1:**
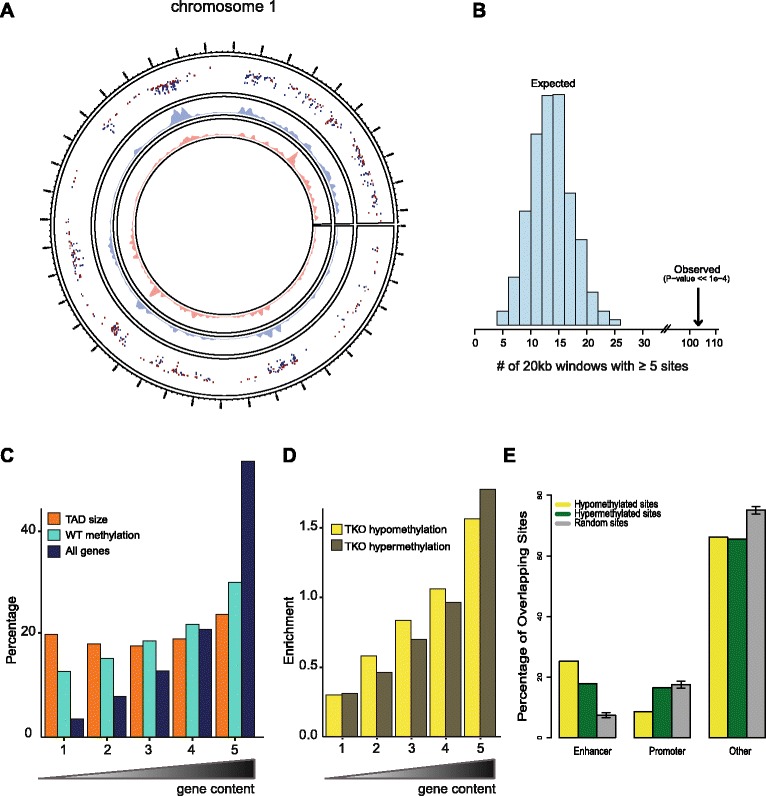
Clustered DNA methylation changes in histone H1-depleted ES cells. **a** Circos plot showing the genome-wide distribution of hypomethylated (in *blue*) and hypermethylated (in *red*) loci along the linear sequence of chromosome 1 in H1 TKO compared with wild-type ES cells, based on the HELP-tagging assay. Inner tracks show local density of respective loci. **b** Histogram of number of genomic windows (fixed size, 20 kb, non-overlapping) that contain at least five randomly chosen sites out of the complete set of roughly one million assayable sites in the HELP-tagging assay. The counts in the histogram sum to the total number of random draws, i.e., 1000. The *arrow* indicates the observed number (103) of genomic windows that contain at least five differentially methylated sites, which is significantly more than expected by chance. **c** Percentages of genes compared with the percentages of DNA methylated sites in wild-type (*WT*) ES cells in groups of TADs ranked according to the number of overlapping genes (*gene content*). The ranking on the x-axis is such that the leftmost bin contains the 20 % TADs with the lowest number of genes and the rightmost bin TADs with the highest number of genes. The genomic sizes of the groups of TADs as a percentage of the total genomic size of all TADs is plotted as a reference. **d** Ratios of both the percentage of hypermethylated or hypomethylated sites in TKO cells over the percentage of DNA methylated sites in WT ES cells in the same groups of TADs as defined before (Fig. 1d). The ratio thus measures the amount of enrichment or depletion of hyper- and hypomethylation in TKO cells in each bin. **e** Spatial distribution of genomic sites where either hypo- or hypermethylation is observed in H1-depleted cells. We analyzed the location of differentially methylated sites with respect to different types of chromatin defined by the ChromHMM algorithm (based on a large collection of mouse ES cell ChIP-Seq data from the ENCODE consortium). For comparison, we include the same distribution for a random selection of sites where DNA methylation status was determined in the HELP-tagging experiment

Local DNA demethylation was previously observed in the H1-depleted TKO ES cells particularly at the imprinting control regions of H19-Igf2 and Gtl2-Dlk1 loci [12] and at the Rhox 5 promoter on the X chromosome [14]. The activity of H1 in promoting DNA methylation at the imprinting control regions was later attributed to H1’s function in recruiting the DNA methyltransferases DNMT1 and DNMT3B [13]. No global changes in DNA methylation were observed at that time, based on digestion with methylation-sensitive restriction enzymes [12]. To study genome-wide methylation changes in more detail, here we used the HELP-tagging assay that enables high-throughput identification of sequences neighboring restriction sites of the methylation-sensitive HpaII enzyme [23]. Approximately 15,000 sites displayed differential methylation across the genome. Consistent with a function of H1 in recruiting DNA methyltransferases [13], more than two-thirds of these sites appeared hypo-methylated in TKO cells, while ~30 % were hypermethylated. Hypermethylation often was not as pronounced as hypomethylation though, and when more stringent criteria were applied for selecting differentially methylated regions (DMRs), 4315 hypomethylated sites were found versus only 308 hypermethylated sites (93 % versus 7 % of all DMRs). The differentially methylated sites were not uniformly distributed across the genome but appeared significantly clustered (Fig. 1a, b), with the sex chromosomes being surprisingly protected against methylation changes (Figure S1 in Additional file 1). To further delineate the clustered distribution, we investigated the density of differentially methylated sites in topologically associating domains (TADs). We considered TADs as chromosomal units of interest as they mark genomic segments within which sequences preferentially contact each other. As such, TADs are believed to be the structural and functional genomic units that encompass the genes and their cognate regulatory sites [15, 24]. We categorized TADs according to gene content and created five bins with equal numbers of TADs; each bin had a similar average TAD size, but the gene-poorest bin encompassed only 3–4 % of all genes while the gene-richest bin carried over 50 % (Fig. 1c). Overall, DNA methylation density followed gene density (Fig. 1c). Taking this distribution into account we found that changes in DNA methylation significantly clustered in the most gene-rich TADs, no matter whether we considered hyper- or hypo-methylation events (Fig. 1d). Thus, bins in gene-dense TADs showed more changes in methylation than bins with equal CpG density in gene-poor TADs (Figure S2 in Additional file 1). Collectively, the data show that DNA methylation changes preferentially occur in chromosomal segments with a high gene density.

To investigate whether the DNA methylation changes associated with H1 depletion localized to any specific type of chromatin, we used published ES cell ChIP-seq profiles and a partitioning of the genome into different chromatin states by means of ChromHMM, a segmentation algorithm for the identification of chromatin states based on the presence of combinations of chromatin modifications [25]. We found that particularly predicted enhancers were strongly overrepresented among the DMRs (Fig. 1e). CpG-rich gene promoters, on the other hand, appeared underrepresented among the hypomethylated sites (Fig. 1e).

In summary, a reduction in the amount of histone H1 protein results in abundant changes in DNA methylation, with some sites gaining but most sites losing methylation. Methylation changes accumulate at the most gene-dense TADs. They occur particularly at enhancer sequences, further indicating that histone H1 plays a role in controlling the DNA methylation status at potential regulatory sequences. In contrast, CpG-rich promoter sequences appear to stably maintain their methylation status in TKO cells, indicating that they control methylation levels in a histone H1-independent manner. A previous observation that H1c and H1d variants are depleted around the transcription start site of active promoters may be in agreement with this idea [26].

### Altered genomic regulatory landscape in H1 TKO cells

**Fig. 2:**
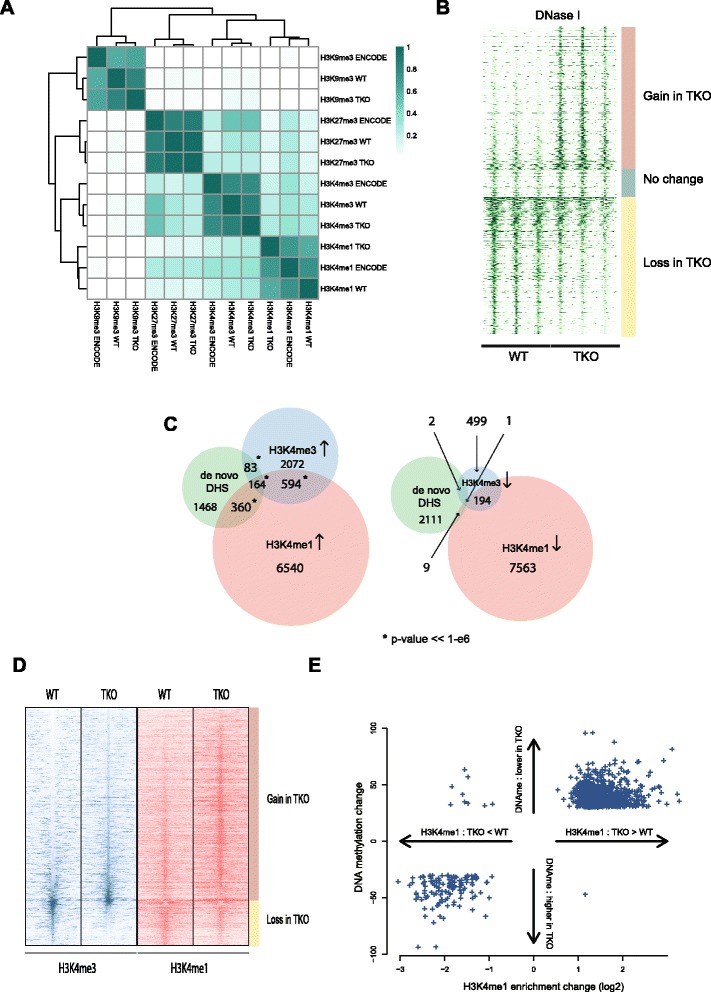
Altered genomic regulatory landscape in H1 TKO cells. **a** Clustered heatmap of fraction of overlap of enriched regions (peaks) in ChIP-sequencing experiments. We compare our ChIP-seq data for the histone modifications H3K4me1, H3K4me3, H3K27me3, and H3K9me3 in wild-type (*WT*) and TKO cells to ChIP-seq data for these marks published by the ENCODE consortium in another mouse ES cell line. **b** Heatmap of DNase-seq coverage in triplicate experiments in WT and H1 TKO cells. Genome-wide statistical analysis of differences in DNase-seq coverage between WT and TKO cells revealed 2123 sites (*top*) with a gain and 2043 sites (*bottom*) with a loss of DNaseI hypersensitivity in TKO cells, respectively. The rows of the heatmap correspond to these ~4000 sites, ordered by log-fold change in coverage, with a random collection of unaltered DNaseI hypersensitive sites in between. **c** Venn diagrams that contain counts of sites that gain (*left*) or lose (*right*) enrichment of the histone marks H3K4me1 and H3K4me3 and their overlap with the 2123 newly formed DHSs in TKO cells. **d** Heatmaps of ChIP-seq enrichment for histone marks H3K4me1 and H3K4me3 in WT and H1 TKO cells. Profiles represent averages over duplicate experiments. Genomic sites represented by rows in the heatmap are sites where significant changes in H3K4me3 enrichment are observed. Rows are ranked by the magnitude of that change from top to bottom in descending order of increase in H3K4me3 enrichment in TKO cells. **e** Scatter plot of changes in H3K4me1 enrichment and DNA methylation at sites where significant changes in both are observed

To further characterize the consequences of histone H1 depletion on the regulatory chromatin landscape of ES cells, we determined the distribution of DNase hypersensitivity sites (DHSs), H3K4me1, H3K4me3, H3K27me3, and H3K9me3 sites across the genome of H1 TKO ES cell lines and their wild-type counterparts. Overall, the histone modification distributions looked similar between the wild-type, TKO cells and those published by ENCODE for another ES cell line (Fig. 2a; Figure S3 in Additional file 1). Roughly identical numbers of DHSs were scored in the two conditions (281,934 in TKO versus 293,319 in wild type, medians over triplicates). When ranked according to their delta DHS signal, de novo formed DHSs were clearly appreciable, but there was little evidence for complete loss of DHSs in H1 TKO cells (Fig. 2b). Chromatin-associated histone H1 was previously found to interfere with the binding of histone methyltransferase SET7/9, thereby preventing methylation of H3K4 [13]. Statistical analysis of ChIP-seq differential enrichment (see “Materials and methods”) revealed no change in the overall numbers of H3K4me1 sites across the genome but did show a high number of sites that gained (6536) or lost (7319) mono-methylation, indicating dynamic changes in this enhancer mark (Figure S4a in Additional file 1). When looking at the H3K4me3 ChIP-seq results we found four times more sites with increased than with decreased levels of trimethyation (2043 versus 495) (Figure S4b in Additional file 1). Surprisingly, compared with the abundant changes observed for the active marks H3K4me1 and H3K4me3, changes in the repressive histone modifications H3K9me3 and H3K27me3 were almost negligible. When using the same fold change cutoffs as used to identify sites with an altered H3K4 methylation status, only a few dozen sites showed dynamic changes in the repressive H3K9me3 and H3K27me3 marks (not shown). A largely unaltered distribution of these repressive marks seems surprising given our earlier finding that histone H1 physically recruits the heterochromatin-specific histone H3 lysine 9 methyltransferase Su(var)3-9 in *Drosophila* [27] but is in agreement with our observations that the intranuclear distribution of histone marks H3K27me3/H3K9me2 and heterochromatin-associated factors such as HP1a, HP1b, and MeCP2 appeared normal by immunofluorescence [12].

We next wished to understand the relationship between these epigenetic changes. Since differences in DHSs were clearest for the 2123 newly formed DHSs, we focused on those DHSs and asked whether their formation coincided with other epigenetic changes. Interestingly, these sites were statistically significantly enriched (Figure S5 in Additional file 1) for the binding motifs of a number of pluripotency factors, including *Klf4* (three-fold enrichment, as judged by HOMER [28]), but also *Oct4* (two-fold) and *Sox2* (two-fold). This suggests that histone H1 normally serves to occlude these sites, which may be in agreement with the earlier observation that wild-type H1 levels are necessary for normal ES cell differentiation and the concomitant repression of *Oct4* expression [29]. Nearly one-third of the new DHSs also showed a gain in either H3K4me1 (*p* < 1e-6, significance of overlap in hypergeometric test) or H3K4me3 (*p* < 1e-6) or both, whereas loss of these marks was very infrequently observed at new DHSs (Figure 2c; Additional file 2). More than 10 % (256/2123) of the new DHSs also revealed loss of DNA methylation, while the opposite, hypermethylation, was rarely found at these sites (19 times) (Figure S6 in Additional file 1). When focusing on H3K4me3 sites, those with increased H3k4me3 levels often (>25 %) also showed a gain (and seldom a loss) in H3K4me1, while sites losing H3K4me3 frequently showed concomitant loss of H3K4me1 (also >25 %) (Fig. 2c). Finally, when considering the differentially methylated CpGs, sites with reduced methylation in TKO cells were often enriched for H3K4me1 marks and, vice versa, hypermethylated sites frequently lost H3K4me1 (Fig. 2e).

In summary, the depletion of histone H1 has little impact on the genome-wide distribution of the repressive histone modifications H3K27me3 and H3K9me3, but changes the active chromatin H3K4me1 and H3K4me3 signatures of thousands of sites across the genome. Many of them show concomitant loss or gain of multiple chromatin marks associated with regulatory activity. Nearly invariably these combinatorial changes are either all positively or all negatively contributing to an active chromatin signature, implying that they can cooperate to strengthen or dampen the regulatory potential of a site.

### Epigenetic changes accumulate in gene-dense TADs

**Fig. 3:**
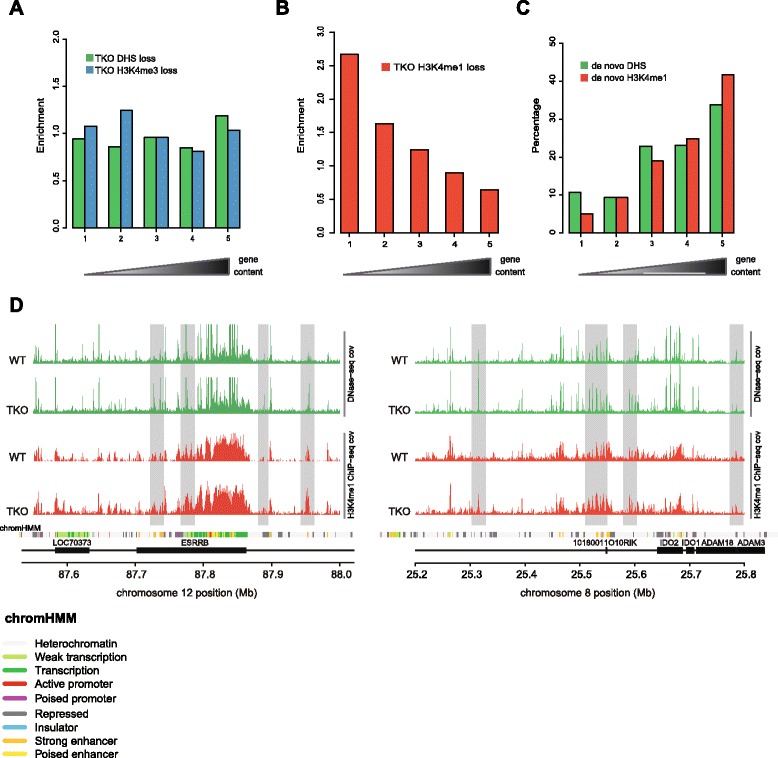
Epigenetic changes accumulate in gene-dense TADs. **a** Ratio of (the percentage of) sites with a significant loss of DHSs in TKO cells, over the (percentage of) DHSs in wild-type (*WT*) ES cells in groups of TADs. The TADs are ordered based on gene content and grouped in equally sized bins (same ordering as in Fig. 1d), with the most gene poor TADs on the left. An analogous ratio is plotted for the sites that lose H3K4me3 in TKOs, but here the ratio is computed relative to the WT sites with H3K4me3 enrichment. **b** Same as panel (**a**), but for sites that significantly lose H3K4me1 enrichment in TKO cells (with the ratio compared relative to the WT H3K4me1 sites). **c** Percentages of de novo DHSs in groups of TADs ranked according to the number of overlapping genes (same ranking as in (**a**, **b**)). Also shown are the percentages of de novo H3K4me1 sites in TKO ES cells. **d** Example of two loci, one on chromosome 12 and one on chromosome 8, where several novel DHSs appear which co-occur with changes in H3K4me1 in TKO ES cells (highlighted in *grey*). Normalized DNase-seq coverage is plotted in *green* (averaged over triplicate experiments in WT and TKO) and normalized H3K4me1 ChIP-seq coverage is plotted in *red* (averaged over duplicates). *Black boxes* indicate genes and a track containing the different computationally predicted chromatin states in WT mouse ES cells (*chromHMM*) is shown as a reference

We then wished to understand where in the genome these epigenetic changes take place. For this we again considered TADs as the genomic units of interest and we intersected the various datasets with the previously defined five classes of TADs. Not surprisingly, the general distribution of DHSs and H3K4me1 and H3K4me3 sites in wild-type (and TKO) cells closely followed that of genes, with all of these marks specifically accumulating in the most gene-dense TADs (Figure S7a in Additional file 1). The same was true for H3K27me3, while H3K9me3 distributed more equally across TADs with different gene density, albeit slightly accumulated in both the most gene-poor as well as the most gene-rich TADs (Figure S7b in Additional file 1). When correcting for their overall distribution there was no obvious enrichment of sites losing DHS or H3K4me3 signal in any of the TAD bins (Fig. 3a). This suggests that sites showing loss of hypersensitivity or loss of the promoter mark H3K4me3 are distributed proportional to the overall genomic localization of DHSs and H3K4me3 sites. In contrast, sites losing H3K4 monomethylation in H1 TKO cells were significantly depleted (chi-squared test *p* < 10^−6^) from the most gene-dense TADs and appeared to accumulate in the gene-poorest TADs (Fig. 3b). This could indicate that normal histone H1 levels are needed for proper maintenance of H3K4me1 levels in inactive chromatin surroundings. Alternatively, H3K4me1 sites in active chromatin surroundings are relatively protected against demethylation. To further investigate if epigenetic alterations occurred at specific genomic locations, we looked at de novo acquired active chromatin marks. We defined de novo DHSs as those that were exclusively identified in TKO cells but also lacked threshold H3K4me1 or H3K4me3 levels in wild-type cells. Similarly, we defined de novo formed H3K4me1 sites as those scoring positive for this mark only in TKO cells and also lacking significant H3K4me3 and DHS signal in wild-type cells. These non-bookmarked sites are ubiquitously present and their conversion into active sites may, therefore, a priori take place anywhere in the genome. However, new DHSs and new H3K4me1 sites both preferentially accumulated again in the most gene-dense TADs (Fig. 3c, d). Thus, despite being a generic chromatin component present throughout the genome, depletion of H1 results in a preferential gain of the active H3K4me1 and H3K4me3 chromatin marks within the most gene-dense TADs. These TADs are already dense in such regulatory chromatin signatures and this, we speculate, may create sensitized chromatin that is exquisitely susceptible to further epigenetic changes. An alternative, not mutually exclusive, explanation is that these TADs form nuclear compartments where the corresponding chromatin modifying enzymes accumulate to cooperatively establish and maintain the required dense landscape of regulatory sites. Perturbing the integrity of the chromatin template, as occurs when H1 histone levels are reduced, may further stimulate local mass action and increase the chance to modify neighboring chromatin sites. Precedence for local cooperative action exists: it was recently shown in *Drosophila* that clustered low affinity binding sites better accumulate PcG proteins than their more isolated counterparts elsewhere in the genome [30].

### Genes with altered expression are proportionally distributed across the genome

**Fig. 4:**
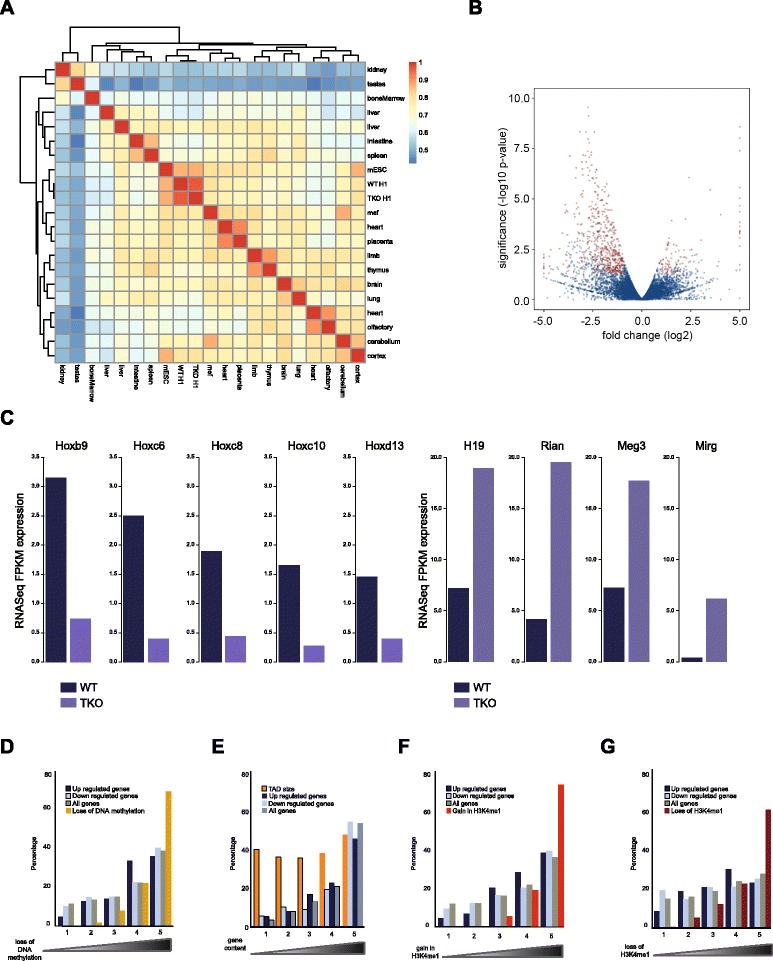
Genes with altered expression are proportionally distributed across the genome. **a** Clustered heatmap of pair-wise correlation between RNA-seq gene expression profiles. We compare RNA-seq gene expression in our wild-type (*WT*) and TKO cells to RNA-seq data from a wide range of mouse tissues published by the ENCODE consortium. *mESC* mouse embryonic stem cell. **b** Volcano plot of statistical significance (-log10 *p* value) against fold change comparing RNA-seq gene expression between WT and H1 TKO mouse ES cells. Transcripts that are significantly differentially expressed between the two conditions are shown in *red*, whereas the genes in *blue* do not reach the threshold. **c** RNA-seq normalized expression values for a selection of transcripts previously reported to be down-regulated (*Hox* genes, *left panel*) and up-regulated (imprinted genes, *right panel*) in H1-depleted ES cells. **d** Percentages of differentially up- and down-regulated genes compared with the percentages of sites with a significant loss of DNA methylation in TKO ES cells and compared with all mouse genes. Percentages are calculated in groups of TADs ranked according to the number of overlapping sites that lose DNA methylation in TKOs. The ranking on the x-axis is such that the leftmost group contains the 20 % TADs with the lowest number of TKO hypomethylated sites and the rightmost group of TADs contains the highest number of such sites. **e** Percentages of differentially expressed genes compared with all genes in groups of TADs ranked according to the number of genes. The ranking on the x-axis is such that the rightmost group contains the 20 % TADs with the highest number of genes and the leftmost group of TADs contains the lowest number. The genomic sizes of the groups of TADs as a percentage of the total genomic size of all TADs is plotted as a reference. **f** Percentages of differentially up- and down-regulated genes compared with the percentages of sites with a significant increase in H3K4me1 enrichment in TKO ES cells and compared with all mouse genes. Percentages are calculated in groups of TADs ranked according to the number of overlapping sites that gain H3K4me1 in TKOs. The ranking on the x-axis is such that the leftmost group contains the 20 % TADs with the lowest and the rightmost group of TADs contains the highest number of these sites. **g** Percentages of differentially up- and down-regulated genes compared with the percentages of sites with a significant decrease in H3K4me1 enrichment in TKO ES cells and compared with all mouse genes. Percentages are calculated in groups of TADs ranked according to the number of overlapping sites that lose H3K4me1 in TKOs. The ranking on the x-axis is such that the leftmost group contains the 20 % TADs with the lowest and the rightmost group of TADs contains the highest number of these sites

To investigate how the altered regulatory chromatin landscape functionally translates into gene expression changes, we assayed the genome-wide transcriptome. RNA-seq confirmed previous observations obtained by microarray analysis. Transcription of the vast majority of genes is not affected in TKOs and the cells still clearly harbor an ES cell identity (Fig. 4a). We again found a small subset of genes (<200), apparently randomly distributed across all chromosomes, that showed a significant twofold or higher change in expression, the majority (>75 %) of which showed reduced levels of transcription (Fig. 4b). Among those were the previously described *Hox* genes [31], while the most prominently upregulated genes included a series of paternally imprinted genes [12] (Fig. 4c). The slight overrepresentation of X-linked genes that was previously apparent among 29 dysregulated genes [12] was no longer appreciable in this larger set of differentially expressed genes. Previous detailed characterization of two of the most strongly upregulated loci in TKO cells, the paternally imprinted *Gtl2* locus and the *H19* locus, revealed hypomethylation of their imprinting control regions [13]. To investigate whether loss of DNA methylation generally underlies transcriptome changes we compared the genomic distribution of up- and down-regulated genes and differentially methylated sites at the level of TADs. To maximally exploit the benefit of an integrative analysis, we considered a less stringent set of 598 differentially expressed genes. We ranked TADs based on the number of DNA de-methylated sites and computed the fractions of differentially regulated genes. Figure 4d shows that indeed TADs with most changes in DNA methylation co-segregated with those most enriched for differentially expressed genes. However, given the non-uniform genomic distribution of differentially methylated sites over gene-dense TADs (Fig. 1d), we considered the overall distribution of genes to be a confounding factor here. To investigate this in more detail we ranked TADs according to gene content. Indeed, this categorization highly correlated with the distribution of differentially expressed genes (Fig. 4e), implying that, from a genomic distribution point of view, they are a proportional and apparently random collection of genes. Possibly in agreement with this, a gene ontology enrichment analysis on the set of differentially expressed genes did not reveal any specific gene ontology categories to be highly enriched. For the sites with changes in DNase I hypersensitivity, the analysis at TAD level is not really appropriate as they are too scarce in individual TADs, so instead we computed the percentages of genes where a significant change in DNase I hypersensitivity occurred within 2500 bp up- or downstream of the transcription start site of the gene. For up-regulated genes, we saw that 6.5 % and 3.2 % had a significant loss and gain, respectively, in DHSs. For down-regulated genes these percentages were 4.6 % and 2.4 %. Hence, the vast majority of differentially expressed genes did not show significant changes in DHSs at their promoters. In cases where changes were found, gain and loss of hypersensitivity was uncorrelated to up- and down-regulated gene activity. The same was true when comparing the distribution of differentially expressed genes to that of TADs with increased H3K4me1 signal (differentially expressed genes strictly followed the overall gene distribution; Fig. 4f), whereas no correlation was observed when comparing differentially expressed genes with TADs with decreased H3K4me1 signal in TKO cells (Fig. 4g).

In summary, while new epigenetic features de novo acquired in H1 TKO cells preferentially appeared in TADs that in wild-type cells already carried the highest density of these marks, these same TADs, when normalized for gene content, were not enriched for deregulated genes. The mere consideration of epigenetic changes is therefore not sufficient to predict gene expression changes. Rather than being dependent on the overall density of epigenetic marks in the TAD, the transcriptional output of individual genes is, therefore, more likely controlled by the regulatory status of only a limited collection of regulatory modules that, possibly via chromatin looping, act on the target gene.

### Higher-order topological changes follow epigenetic but not transcriptional changes

**Fig. 5:**
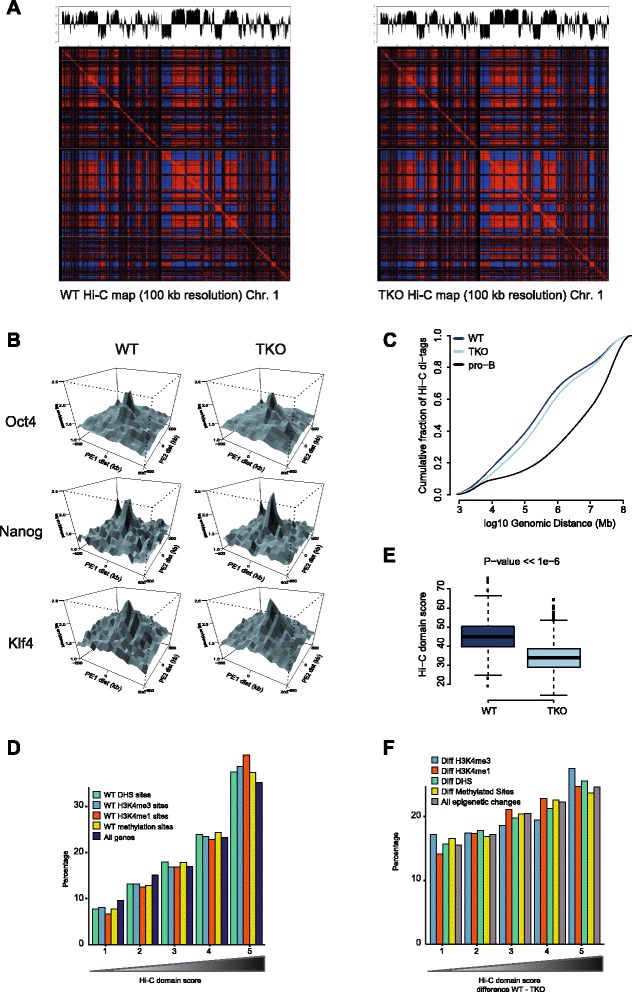
Higher-order topological changes follow epigenetic but not transcriptional changes. **a** Normalized Hi-C interaction heatmap showing chromatin compartmentalization (A/B compartments) at 100 kb resolution for chromosome 1 in wild-type (*WT*; *left*) versus TKO cells (*right*). Coefficients of the first principal component (PC1) of the Hi-C interaction heatmap are plotted on top along the linear sequence of chromosome 1, showing no apparent changes in chromatin compartment organization upon H1 depletion in mouse ES cells. **b** PE-SCAN Hi-C analysis probing Hi-C interactions between clusters of binding sites for transcription factors that control mouse ES cell identity (pluripotency factors). ES cell-specific interactions among binding sites of *Oct4*, *Nanog*, and *Klf4* remain present in mouse ES cells upon depletion of H1 in TKO cells. **c** Plot comparing the distribution of Hi-C interactions versus genomic distance for three different Hi-C maps. Mouse ES cells are characterized by a relatively large fraction of interactions over short distances, whereas differentiation is known to be accompanied by an increase in long-range interactions. The TKO Hi-C map clearly shows a shift towards those of a more differentiated cell. **d** Percentages of genes in groups of TADs ranked according to the Hi-C domain score in WT ES cells. The ranking on the x-axis is such that the leftmost group contains the 20 % TADs with the lowest and the rightmost group of TADs contains the highest Hi-C domain scores. We also show the distribution of sites, in WT ES cells, enriched for the histone marks H3K4me1and H3K4me3, sites with DNA methylation and DHSs in these groups of TADs. **e** Boxplots comparing the Hi-C domain score of all mouse ES cell TADs in our WT and TKO Hi-C maps. A two-sample Wilcoxon rank sum test was applied to test the significance of the shift in domain score in TKO cells (*p* value < < 1e-6). **f** Percentages of the total sum of all epigenetic changes in groups of TADs ranked according to the difference in Hi-C domain score between TKO and WT ES cells. The ranking on the x-axis is such that the leftmost group contains the 20 % TADs with the lowest and the rightmost group of TADs contains the highest difference in Hi-C domain score. We also show the percentages for the individual changes in the histone marks H3K4me1and H3K4me3, differential DNA methylation and differential DNase I hypersensitivity

We finally wished to understand how histone H1 depletion and the accompanying epigenome and transcriptome changes affect the overall 3D organization of the genome. To this end we performed replicate Hi-C experiments, each with a different, frequently cutting, restriction enzyme (NlaIII and DpnII) [32], in both TKO and matched wild-type ES cells. Each dataset contained between 26 and 42 million valid Hi-C read pairs, adding up to 53 milliom (wild-type) and 76 million (TKO) valid Hi-C read pairs per cell type. All Hi-C libraries showed an equal high ratio of intra- over inter-chromosomal contacts (~75 %), indicative of good quality Hi-C libraries [33]. We normalized and processed Hi-C data by binning reads per 100 kb chromosomal segments to generate contact heatmaps as described before [22]. Visual inspection of the heatmaps suggested that chromosomes folded very similar between wild-type and TKO cells (Fig. 5a). Principal component analysis of Hi-C data was previously used to uncover an A and B compartment where active and inactive chromatin regions, respectively, preferentially cluster. When applied to our datasets it showed that chromosomal domain organization and overall 3D genome structure is indeed very similar between the two cell types (Fig. 5a). In fact, the contact profiles that we generated for wild-type and histone H1-depleted ES cells were more similar to each other than any of the two was to a previously published wild-type ES cell contact profile [15] or pro-B-cell contact profile [34] (Figure S8 in Additional file 1). Thus, a 50 % depletion of linker histone H1 can be tolerated without profound changes in the overall 3D genome.

We previously reported that ES cells harbor a unique 3D genome, hallmarked in general by a more random higher order topology with particularly the inactive chromatin compartment being spatially unorganized. Another feature of the pluripotent 3D genome is the specific clustering of genomic regions dense in binding of pluripotency factors [21]. This preferential clustering of dense pluripotency factor-associated regions was also appreciable in both our wild-type and H1 TKO cells (Fig. 5b) and confirmed that the TKO cells harbored an ES cell identity (Fig. 5c). Compared with their wild-type counterparts, the TKO cells showed a slightly increased capacity for chromosomal regions to contact each other over distance, but this effect clearly was not as pronounced as seen in, for example, differentiated pro-B cells (Fig. 5c).

Realizing that the overall topology of chromosomes is unaltered in H1-depleted cells but that domains gain some capacity to reach out and contact other domains elsewhere on their chromosome, we used the recently published TAD cross-boundary ratio to search for more subtle topological changes. The TAD cross-boundary ratio divides the intra-domain contacts over the inter-domain contacts [35] and as such can serve as a domain score. It is the most gene-dense TADs that are most enriched for DHSs and for active chromatin marks which show the highest such domain score (Fig. 5d). Active TADs therefore seem the structurally most isolated chromosomal entities. We calculated this value for each TAD in wild-type and H1 TKO cells and compared them. In agreement with the observation that in histone H1 depleted cells chromosomal sites more easily engage in contacts over very large distances, TADs generally had a lower domain score in TKO versus wild-type cells (Fig. 5e). Histone H1 therefore seems to contribute to the topological segmentation of chromosomes.

We then wished to identify the TADs that were most sensitive to topological changes upon histone H1 depletion. For this we computed the difference in domain score between wild-type and TKO cells and ranked TADs accordingly. Despite almost all TADs showing a reduction in the domain score and despite this difference being relatively modest, we nevertheless found that the degree of structural changes significantly correlated (chi-squared test *p* < < 10^−6^) with the amount of epigenetic changes observed per TAD (Fig. 5f). This was true no matter which of the investigated marks was considered. Thus, the top 20 % topologically most altered TADs were those that also carried most sites with altered hypersensitivity, most sites with modified levels of H3K4me1 and/or H3K4me3 and most differentially methylated CpGs. In contrast, the TADs most resistant to topological changes were those showing the least epigenetic changes upon H1 depletion. Interestingly, such correlations were not found with gene density, TAD size or differential gene expression (Figure S9 in Additional file 1). It is, therefore, not necessarily the TADs with highest gene content nor the larger TADs and also not the TADs with most striking changes in transcriptional output that are most sensitive to topological changes. Rather, alterations in the epigenetic landscape appear to best correlate with topological alterations of TADs. Thus, while gene expression clearly correlates with the nuclear positioning of TADs relative to each other and to, for example, the nuclear periphery, our current data provide further evidence that gene expression and higher-order chromosome topology are not causally related [36–38]. Rather, they may be independently controlled by the locally associated *trans*-acting factors.

## Conclusions

Our data show that cells require normal histone H1 levels to expose their proper regulatory landscape. Reducing the levels of histone H1 results in massive epigenetic changes and altered topological organization, particularly at the most active chromosomal domains. Changes in TAD configuration coincide with epigenetic landscape changes but not with transcriptional output changes, supporting the emerging concept that transcriptional control and nuclear positioning of TADs are not causally related but independently controlled by the locally associated *trans*-acting factors.

## Materials and methods

### Cell culture

Wild-type and H1 TKO ES cells [12] were grown on irradiated mouse embryonic fibroblasts in Dulbecco's modified Eagle's medium (high glucose, Gibco) with 15 % fetal bovine serum, 1× non-essential amino acids (NEAA; Gibco), 1× penicillin–streptomycin (Gibco), 1:1000 b-mercaptoethanol (Invitrogen), 1× L-glutamine (Gibco) and 1000 U/ml leukaemia inhibitory factor (Gibco).

### Hi-C template

Cells were trypsinized and plated on uncoated plates for 30 minutes at 37 °C to get rid of the feeder cells. Then a 3C template was generated as previous described [39]. In brief, 10 million cells were cross-linked by 2 % formaldehyde, then digested with DpnII or NlaIII, and ligated to form 3C circles. Purified 3C products were then further sheared to 600–800 bp. Sheared DNA (1 μg)was used to generate high-throughput sequencing-ready sample by using TruSeq DNA sample prep kit (Illumina), following the standard commercial protocol. The Hi-C library was sequenced with Illumina paired-end sequencing.

### ChIP-seq

Chromatin immunoprecipitation (ChIP) was performed as described previously [12, 13] with ChIP grade H3K4me1- and H3K4me3-specific antibodies purchased from Abcam with a few modifications. Fixed cells were lysed in a buffer containing 10 mM Na-butyrate and isolated chromatin was sonicated to 500–800 bp with a Covaris S2 sonicator at 4 °C. ChIP-seq library preparation and sequencing were performed by the Epigenomics Core Facility at the Albert Einstein College of Medicine using on an Illumina 2500 HiSeq instrument. We generated duplicate ChIP-seq libraries (both duplicate input and immunoprecipitation samples, with antibodies against H3K4me1 and H3K4me3) for both conditions (wild type and TKO). For the H3K9me3 and H3K27me3 ChIP-seq experiments, 40 million cells of both conditions (wild type and TKO) were cross-linked, washed and lysed as described previously [40]. The obtained nuclei were dissolved in 80 μl sonication buffer, transferred to microtubes and sonicated for 12 cycles of 60 seconds using microtubes in the Covaris S series with the following settings: intensity 3, duty cycle 20 %, 200 cycles/bursts. Supernatant was cleared [40] and added to DynaI protein G beads that were pre-incubated with ab6002 for H3K27me3 and ab8898 for H3K9me3 from Abcam. After immunoprecipitation, beads were washed and DNA was eluted, reverse cross-linked and further purified as described before [40]. ChIP-seq libraries were made according to the Illumina Truseq DNA library protocol, and sequencing was performed at the Utrecht Sequencing Facility on a NextSeq500. Reads from all different libraries where aligned to the reference genome (NCBI37/mm9) with bowtie2 [41] with default settings and the --qc-filter switch. Duplicates were marked using Picard (http://broadinstitute.github.io/picard/) and were removed from the data for subsequent analyses. Regions significantly enriched for H3K4me1, H3K4me3, H3K27me3, and H3K9me3 compared with matched input samples were identified using the MACS2 peak caller [42] with default settings. For the H3K9me3 and H3K27me3 histone marks, the parameter - - broad was set. Analysis of differential ChIP enrichment was done using diffReps [43] with parameters -me gt --pval 0.001 --frag 150.

### RNA-seq

Total RNAs were prepared from ES cells adapted to gelatinized dishes using RiboPure RNA purification kits (Ambion). Paired-end library construction was performed using Tru-seq kits (Illumina). Resulting libraries were run on the Hi-seq 2000 (Salk Institute), generating 2× 100-bp paired-end reads. We aligned reads of two replicate wild-type ES cell RNA-seq libraries and three replicate H1 TKO ES cell RNA-seq libraries to the reference genome (NCBI37/mm9) with TopHat [44] and used Cufflinks and CuffDiff [45] for differential expression analysis of RNA-seq expression for a non-redundant collection of 20,876 known RefSeq transcripts. We considered genes with a marginal *p* value smaller than 0.05 and an absolute log2 fold-change bigger than 1 to be differentially expressed (598 genes).

### Genome-wide DNA methylation analysis using the HELP-tagging assay

Genomic DNA was isolated and digested with HpaII and MspI, and Illumina library preparation was performed exactly as described previously [23]. Library sequencing was performed in the Epigenomics Core Facility at the Albert Einstein College of Medicine. We computed the HELP angle as described in [23] and used it as a measure for the percentage of methylated cytosines. We performed binomial tests for differential methylation and this resulted in 15,492 differentially methylated sites with a *p* value smaller than 1e-6.

### DNase I hypersensitivity assay

DNase I hypersensitivity assay was essentially carried out as described in [46]. In brief, nuclei were extracted in lysis buffer (15 mM Tris‐HCl, 15 mM NaCl, 60 mM KCl, 1 mM EDTA, 0.5 mM EGTA, 0.5 mM spermidine) by incubating for 10 minutes on ice. Then, nuclei were incubated for 3 minutes at 37 °C in the same lysis buffer with 1 mM CaCl_2_ and with limiting concentrations of the DNA endonuclease deoxyribonuclease I (DNase I). Reactions were stopped by adding stop buffer (50 mM Tris‐HCl, 100 mM NaCl, 0.1 % SDS, 100 mM EDTA, 1 mM spermidine, 0.5 spermine, pH 8.0) and purified fragments were recovered by sucrose ultracentrifugation, end‐repaired and ligated with adapters, followed by sequencing on the Illumina sequencing platform. From an initial collection of 33 different DNase-seq libraries (17 wild type, 16 TKO), we filtered out three high quality replicates in each condition according to their SPOT score. Reads were aligned to the reference genome (NCBI37/mm9) and we considered 36-bp reads that aligned uniquely and contained no more than two mismatches as properly mapped reads. We used the Hotspot [47] algorithm to identify DHSs in all six samples separately. For differential DNase-seq analysis between wild type and TKO, we used the PoissonSeq R package [48]. We compared DNase-seq coverage in 89,875 different regions (with sufficient coverage in either condition) and this resulted in a set of 4166 regions with statistically significant difference in coverage after applying a multiple testing procedure (false discovery rate 5 %).

### Statistical analysis

All statistical analyses were performed under R/Bioconductor [49] using custom R scripts. Manipulation with and computation of statistics on genomic intervals and domains was done using the GenomicRanges package [50]. Analysis of ChIP-seq data and DNase I hypersensitivity data and generation of heatmaps were done using the compEpiTools package (http://genomics.iit.it/groups/computational-epigenomics.html).

### Ethical approval

The authors state that no ethical approval was required for this study.

### Availability of supporting data

All the raw and processed data for the experiments described in this paper have been submitted to NCBI Gene Expression Omnibus (GEO) under accession number GSE75426.

## Additional files

Additional file 1: Figure S1. DNA methylation changes per chromosome in histone H1-depleted ES cells. **Figure S2.** Hypomethylation in TKO cells occurs preferentially in gene-dense TADs regardless of GC content. **Figure S3.** ChIP-seq of four different histone marks in wild-type and TKO mouse ES cells. **Figure S4.** Large-scale changes in H3K4me1 and H3K4me3 sites upon depletion of histone H1. **Figure S5.** Analysis of DNA binding motifs at de novo formed DHSs in TKO cells. **Figure S6.** Over 10 % of de novo formed DHSs also show loss in CpG methylation in H1 TKO cells. **Figure S7.** Active chromatin marks accumulate in the most gene-dense TADs. **Figure S8.** Higher-order genome topology is very similar between wild-type and histone H1 TKO cells. **Figure S9.** Changes in compartment organization are not related to gene content, TAD size or differential expression. (PDF 4637 kb) Additional file 2: Table S1. The number of loci at which changes are observed for various combinations of epigenetic marks. (XLS 13 kb)

## Abbreviations

3D: Three-dimensional
bp: Base pair
ChIP: Chromatin immunoprecipitation
DHS: DNase I hypersensitivity site
DMR: Differentially methylated region
ES: Embryonic stem
TAD: Topologically associating domain
TKO: triple knock-out
