# Supplementary material for: Local compartment changes and regulatory landscape alterations in histone H1-depleted cells
Source: Genome Biol. 2015 Dec 23;16:289. doi: 10.1186/s13059-015-0857-0 (PMC4699363; doi:10.1186/s13059-015-0857-0)
Supplement: Additional file 1: Figure S1. — DNA methylation changes per chromosome in histone H1-depleted ES cells. Figure S2. Hypomethylation in TKO cells occurs preferentially in gene-dense TADs regardless of GC content. Figure S3. ChIP-seq of four different histone marks in wild-type and TKO mouse ES cells. Figure S4. Large-scale changes in H3K4me1 and H3K4me3 sites upon depletion of histone H1. Figure S5. Analysis of DNA binding motifs at de novo formed DHSs in TKO cells. Figure S6. Over 10 % of de novo formed DHSs also show loss in CpG methylation in H1 TKO cells. Figure S7. Active chromatin marks accumulate in the most gene-dense TADs. Figure S8. Higher-order genome topology is very similar between wild-type and histone H1 TKO cells. Figure S9. Changes in compartment organization are not related to gene content, TAD size or differential expression. (PDF 4637 kb) [file 13059_2015_857_MOESM1_ESM.pdf]

Supplemental Figure S1

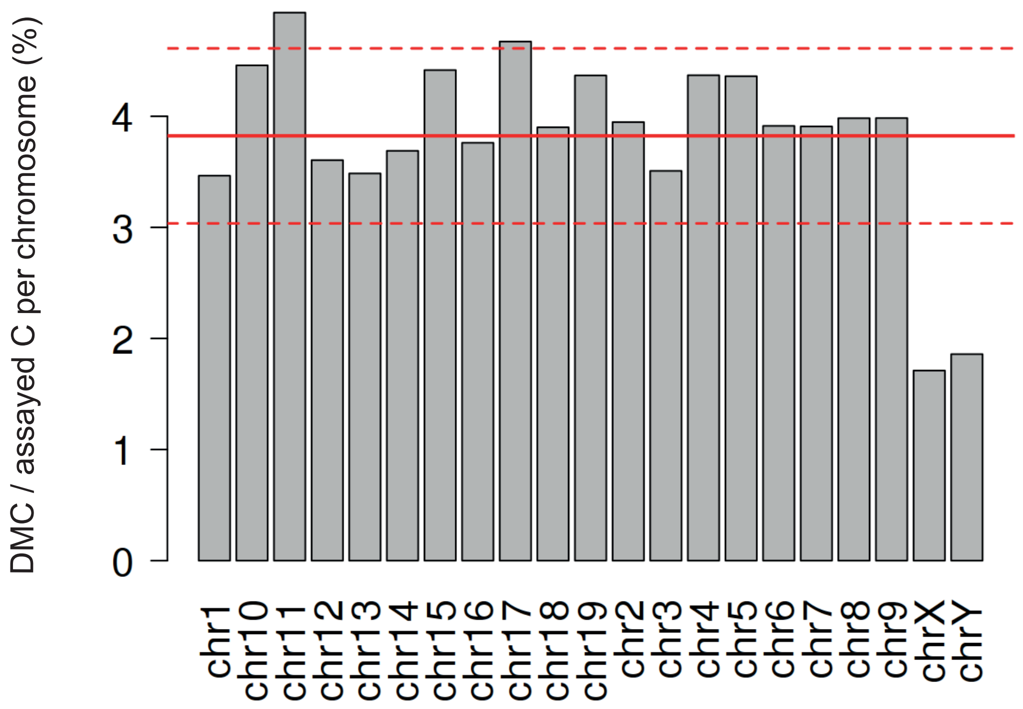

Supplemental Figure 1. DNA methylation changes per chromosome in histone H1 depleted ES cells.

Barplots showing the percentage of differentially methylated cytosine (DMC) per million of assayed cytosines for each chromosome. The red solid and dashed lines mark the mean and the points one standard-deviation away from the mean.

Supplemental Figure S2

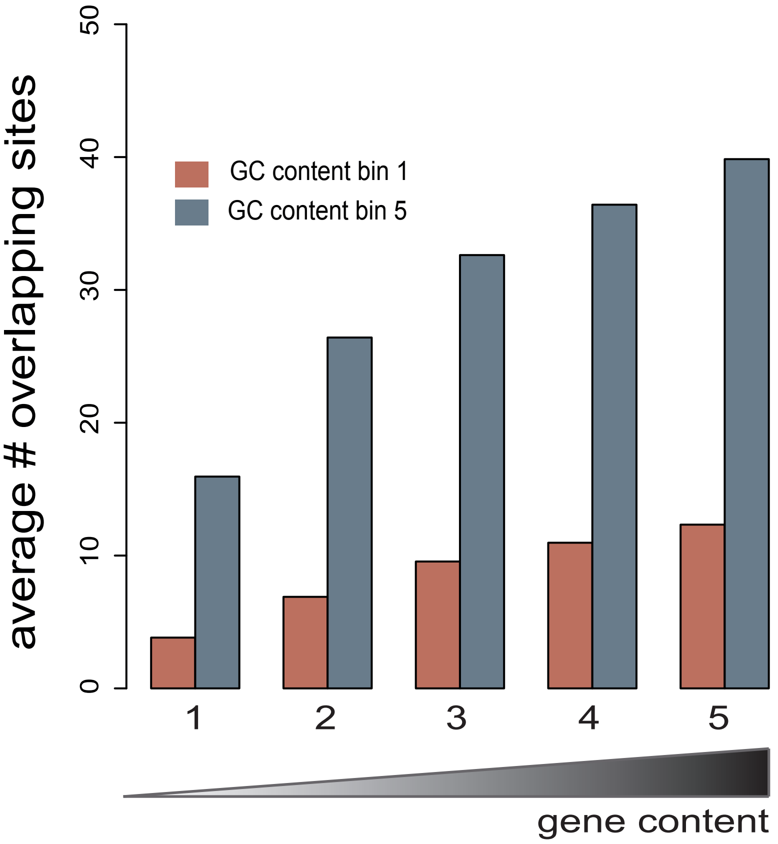

Supplemental Figure S2. Hypomethylation in TKO cells occurs preferentially in gene dense TADs regardless of GC content

We created 1000bp bins within all 2200 TADs and computed the GC content of all these bins. When ranking the TADs according to gene content (as described in the main manuscript), the overall distribution of GC content of these bins is different across the different TADs, with a general increase in average GC content in more gene dense TADs. To determine whether the increase in the number of hypomethylated sites in gene dense TADs simply follows GC density, we decided to do a stratified sampling of 1000bp bins, so that we sample bins with equal GC content from the different TADs. The bins we sample either follow the GC content distribution of 1000bp bins in gene poor TADs (pink, GC content of TAD bin 1) or gene dense TADs (blue, GC content of TAD bin 5). The barplot shows the average number of overlapping hypomethylated sites in 5000 randomly sampled bins.

Supplemental Figure S3

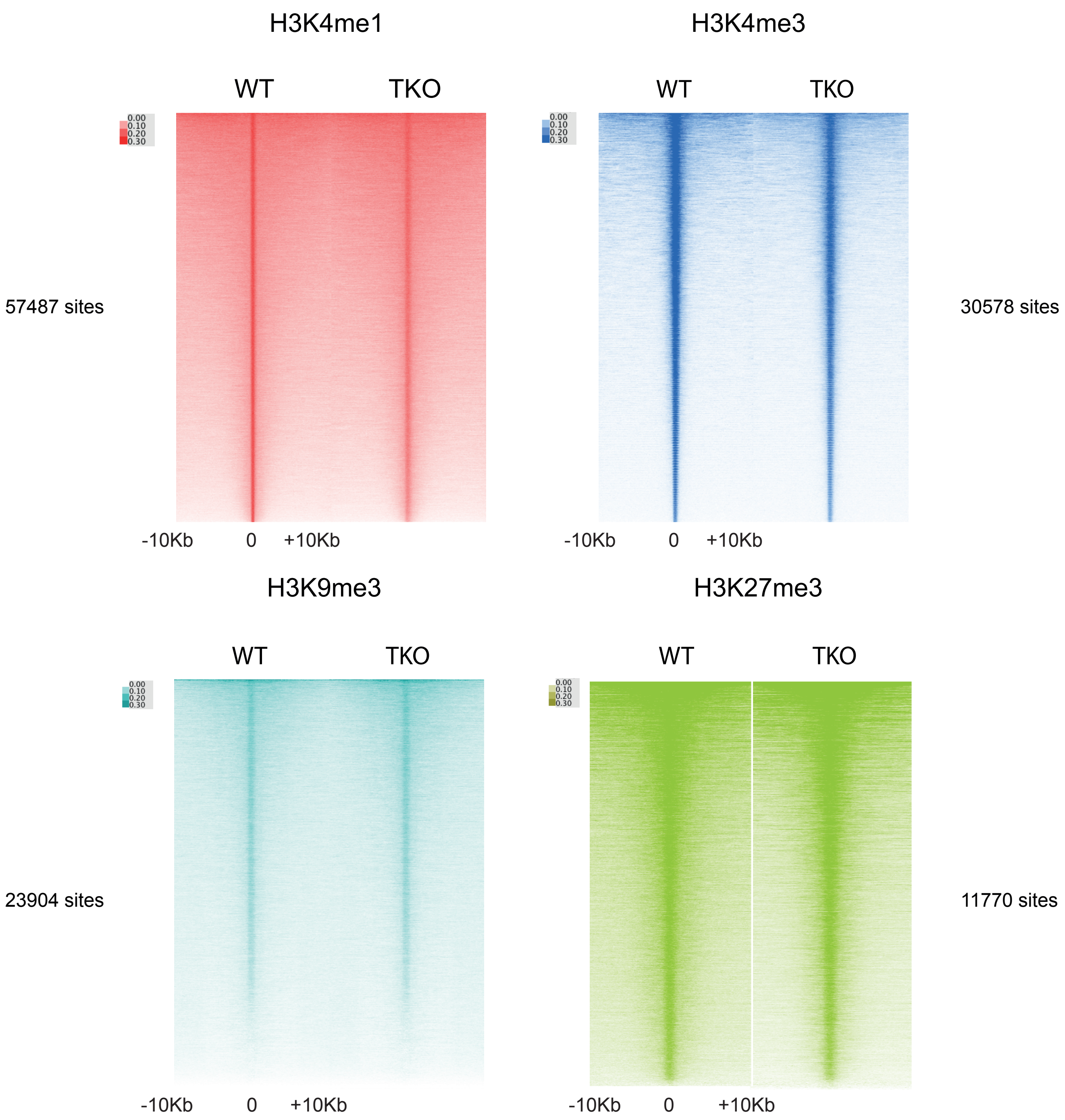

Supplemental Figure S3. ChIP-Sequencing of four different histone marks in WT and TKO mouse ES cells

Heatmaps are shown of sequencing-depth normalized ChIP-Seq coverage in individual IP samples, i.e. not normalized for input, at enriched regions in WT cells for each histone mark separately. Rows in the heatmap represent peaks identified by the MACS2 peak caller and coverage is calculated in 100bp bins, normalized for the total number of mapped reads in each library. Regions where significant differences in ChIP-Seq enrichment are detected are excluded from the heatmaps.

Supplemental Figure S4

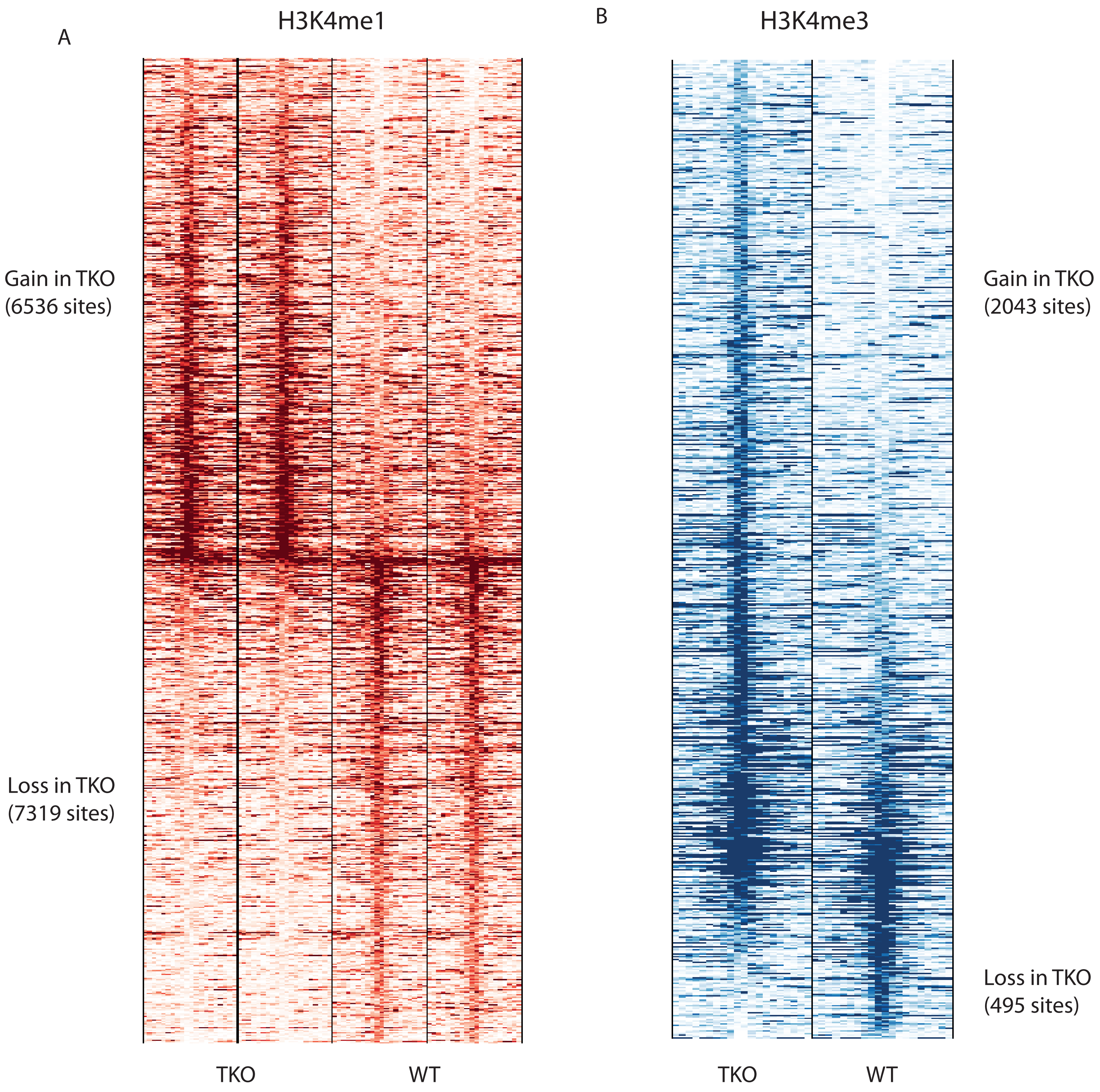

Supplemental Figure S4 Large-scale changes in H3K4me1 and H3K4me3 sites upon depletion of histone H1

Heatmaps of ChIP-seq enrichment for histone marks H3K4me1 and H3K4me3 in wildtype and H1 TKO cells. Genomic sites represented by rows in the heatmap are sites where significant changes in H3K4me1 (A) and H3K4me3 (B) enrichment are observed. Rows are ranked by the magnitude of that change from top to bottom.

Supplemental Figure S5

Rank	Motif	P-value	log P-pvalue	% of Targets	% of Background	STD(Bg STD)	Best Match/Details
1	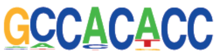	1e-152	-3.516e+02	31.56%	10.49%	51.7bp (60.4bp)	Klf4(Zf)/mES-Klf4-ChIP-Seq(GSE11431)/Homer(0.897) More Information | Similar Motifs Found
2	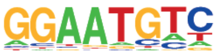	1e-35	-8.065e+01	31.75%	20.25%	53.5bp (58.3bp)	TEAD4(TEA)/Tropoblast-Tead4-ChIP-Seq(GSE37350)/Homer(0.905) More Information | Similar Motifs Found
3	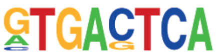	1e-34	-7.892e+01	9.09%	3.32%	54.8bp (59.8bp)	MA0477.1_FOSL1/Jaspar(0.965) More Information | Similar Motifs Found
4	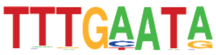	1e-26	-6.209e+01	9.94%	4.36%	52.8bp (57.5bp)	Oct4(POU,Homeobox)/mES-Oct4-ChIP-Seq(GSE11431)/Homer(0.811) More Information | Similar Motifs Found
5	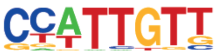	1e-22	-5.074e+01	10.65%	5.28%	53.3bp (59.6bp)	Sox2(HMG)/mES-Sox2-ChIP-Seq(GSE11431)/Homer(0.964) More Information | Similar Motifs Found
6	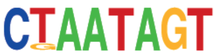	1e-18	-4.244e+01	2.03%	0.35%	50.0bp (58.7bp)	PB0135.1_Hoxa3_2/Jaspar(0.701) More Information | Similar Motifs Found
7	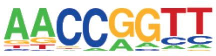	1e-15	-3.473e+01	4.10%	1.53%	49.1bp (56.0bp)	PB0077.1_Spdef_1/Jaspar(0.686) More Information | Similar Motifs Found

Supplemental Figure S5. Analysis of DNA binding motifs at *de novo* formed DHS sites in TKO cells.

Table showing the result of a computational analysis of over-representation of DNA binding motifs at the 2123 *de novo* formed DHS sites in TKO cells. Graphical representations of identified motif sequences are shown together with statistics relating to the size and significance of enrichment in the target set (de novo DHS sites) compared to a background set of WT DHS site sequences. The last column shows matches to known TF binding motifs.

Supplemental Figure S6

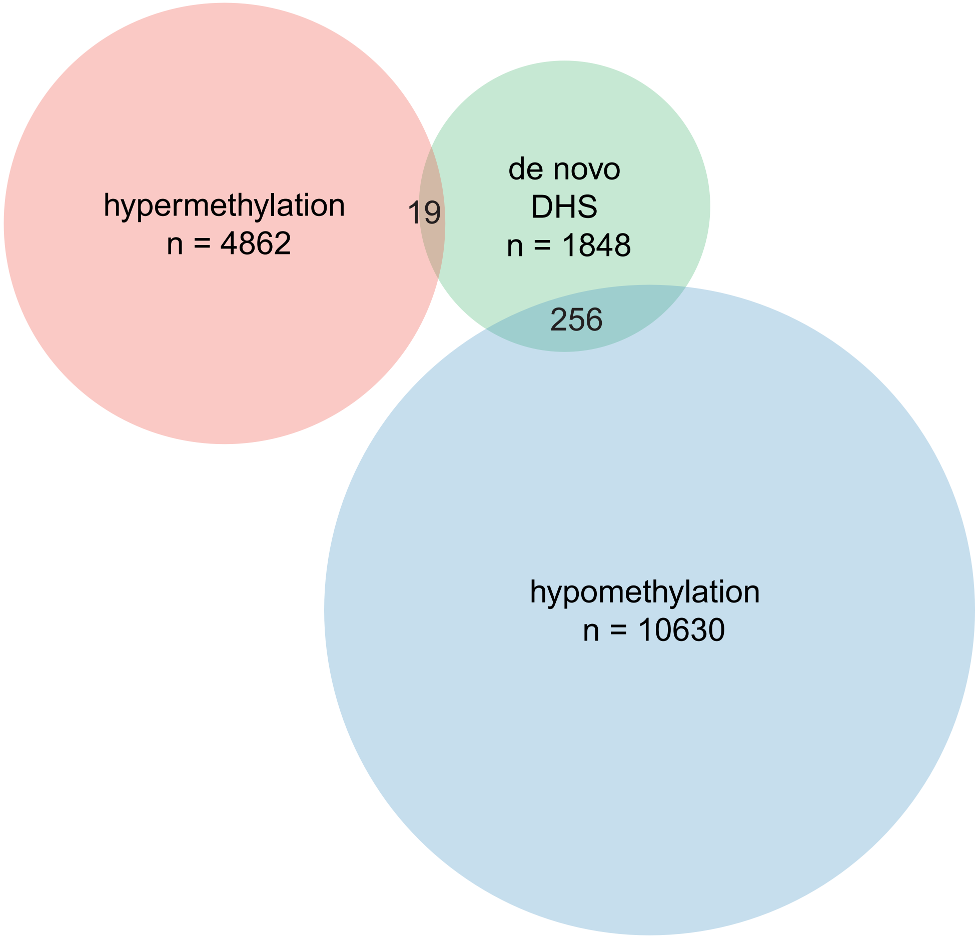

Supplemental Figure S6. Over ten percent of the de novo formed DHS also show loss in CpG methylation in H1 TKO cells.

Venn diagram showing counts of de novo formed DHS and their overlap with sites that show a significant gain or loss of DNA methylation in TKO.

Supplemental Figure S7

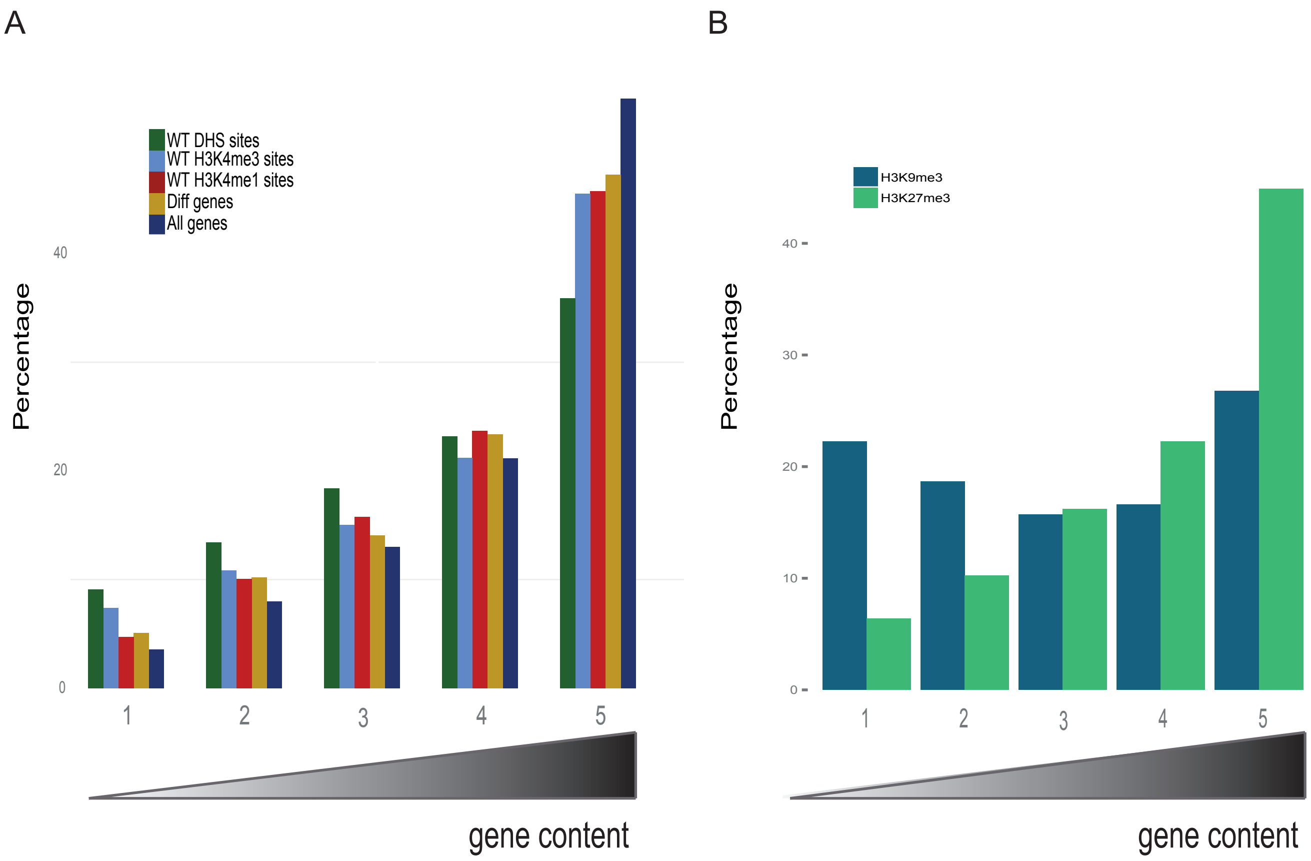

Supplemental Figure S7. Active chromatin marks accumulate in the most gene-dense TADs

Barplots showing the percentages of differentially expressed genes compared to all genes in groups of TADs ranked according to the number of genes contained within a TAD. Also shown is the distribution of sites enriched for H3K4me1, H3K4me3, DNaseI hypersensitive sites (panel A) and sites enriched for H3K27me3 or H3K9me3 (panel B) in WT ES cells over these 5 categories of TADs.

Supplemental Figure S8

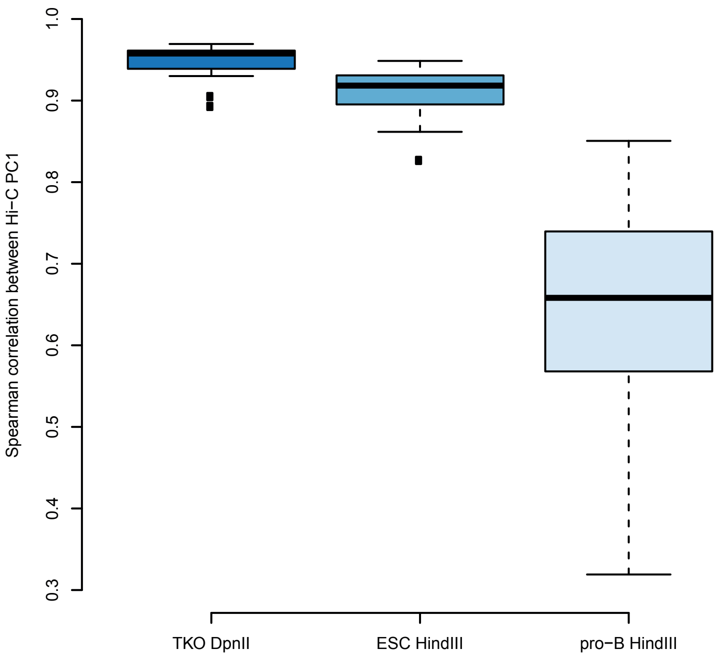

Supplemental Figure S8. Higher-order genome topology is very similar between wildtype and histone H1 TKO cells

The coefficients of the first principal component of the normalized Hi-C interaction matrix can be used to distinguish between the A and B chromatin compartments (see Figure 5A). By comparing the correlation of these coefficients (per chromosome) between conditions, we can measure how similar they are in terms of A and B compartmentalization. Figure S7 shows boxplots comparing the correlation (per chromosome) of PC1 coefficients between our WT DpnII Hi-C map, and the Hi-C maps indicated on the x-axis, i.e. TKO DnpII, ESC HindIII and pro-B HindIII. This shows that in terms of A and B compartment organization, the TKO Hi-C map is more similar to the WT Hi-C map than to a previously published mouse ES Hi-C map (small difference) and to a Hi-C map published in a completely different cell type (large difference).

Supplemental Figure S9

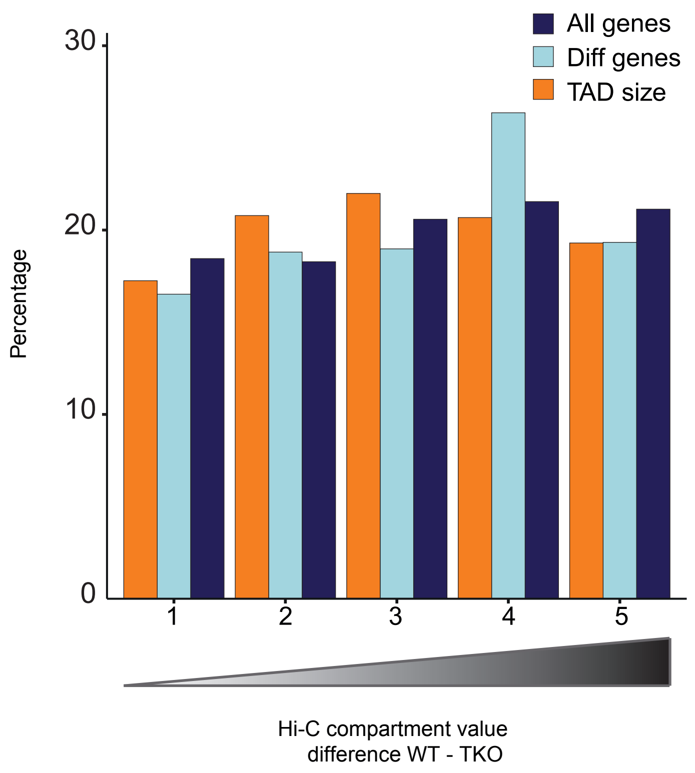

Supplemental Figure S9. Changes in compartment organization are not related to gene content, TAD size or differential expression

Barplots showing the percentages of genes in 5 equally sized groups of TADs ranked according to the difference in Hi-C compartment value between WT and H1 TKO ES cells. Also shown is the distribution of differentially expressed genes over these 5 groups of TADs. The total genomic size of the TADs in each group as a percentage of the total genomic size of all TADs is plotted as a reference.
